# Common Effects on Cancer Cells Exerted by a Random Positioning Machine and a 2D Clinostat

**DOI:** 10.1371/journal.pone.0135157

**Published:** 2015-08-14

**Authors:** Benjamin Svejgaard, Markus Wehland, Xiao Ma, Sascha Kopp, Jayashree Sahana, Elisabeth Warnke, Ganna Aleshcheva, Ruth Hemmersbach, Jens Hauslage, Jirka Grosse, Johann Bauer, Thomas Juhl Corydon, Tawhidul Islam, Manfred Infanger, Daniela Grimm

**Affiliations:** 1 Department of Biomedicine, Aarhus University, Aarhus, Denmark; 2 Clinic for Plastic, Aesthetic and Hand Surgery, Otto-von-Guericke-University Magdeburg, Magdeburg, Germany; 3 DLR, German Aerospace Center, Institute of Aerospace Medicine, Cologne, Germany; 4 Department of Nuclear Medicine, University of Regensburg, Regensburg, Germany; 5 Max-Planck Institute for Biochemistry, Martinsried, Germany; Osaka University Graduate School of Medicine, JAPAN

## Abstract

In this study we focused on gravity-sensitive proteins of two human thyroid cancer cell lines (ML-1; RO82-W-1), which were exposed to a 2D clinostat (CLINO), a random positioning machine (RPM) and to normal 1*g*-conditions. After a three (3d)- or seven-day-culture (7d) on the two devices, we found both cell types growing three-dimensionally within multicellular spheroids (MCS) and also cells remaining adherent (AD) to the culture flask, while 1*g*-control cultures only formed adherent monolayers, unless the bottom of the culture dish was covered by agarose. In this case, the cytokines IL-6 and IL-8 facilitated the formation of MCS in both cell lines using the liquid-overlay technique at 1*g*. ML-1 cells grown on the RPM or the CLINO released amounts of IL-6 and MCP-1 into the supernatant, which were significantly elevated as compared to 1*g*-controls. Release of IL-4, IL-7, IL-8, IL-17, eotaxin-1 and VEGF increased time-dependently, but was not significantly influenced by the gravity conditions. After 3d on the RPM or the CLINO, an accumulation of F-actin around the cellular membrane was detectable in AD cells of both cell lines. IL-6 and IL-8 stimulation of ML-1 cells for 3d and 7d influenced the protein contents of ß_1_-integrin, talin-1, Ki-67, and beta-actin dose-dependently in adherent cells. The ß_1_-integrin content was significantly decreased in AD and MCS samples compared with 1*g*, while talin-1 was higher expressed in MCS than AD populations. The proliferation marker Ki-67 was elevated in AD samples compared with 1*g* and MCS samples. The ß-actin content of R082-W-1 cells remained unchanged. ML-1 cells exhibited no change in ß-actin in RPM cultures, but a reduction in CLINO samples. Thus, we concluded that simulated microgravity influences the release of cytokines in follicular thyroid cancer cells, and the production of ß_1_-integrin and talin-1 and predicts an identical effect under real microgravity conditions.

## Introduction

Prolonged spaceflights often cause deleterious health problems in humans. A number of spaceflight effects have been extensively studied in the past and reviewed [1–4]. Some effects may be explained by well-known physiology; e.g. the lack of gravitational stress on the leg musculature results in a rapid loss of bone and muscle, and the lack of the gravitational vector causes problems related to balance and eye movements [1]. It had been shown that annulling of gravity influences the molecular mechanisms of the cells directly [3]. Cells exposed to real or simulated microgravity change their gene and protein expression behavior [5–7], increase apoptosis [8, 9], retard cell growth [10] and alter the cytoskeleton [11–13]. Moreover, multicellular aggregates were detected, which resembled the organs from which their cells had been derived [14].

**Fig 1:**
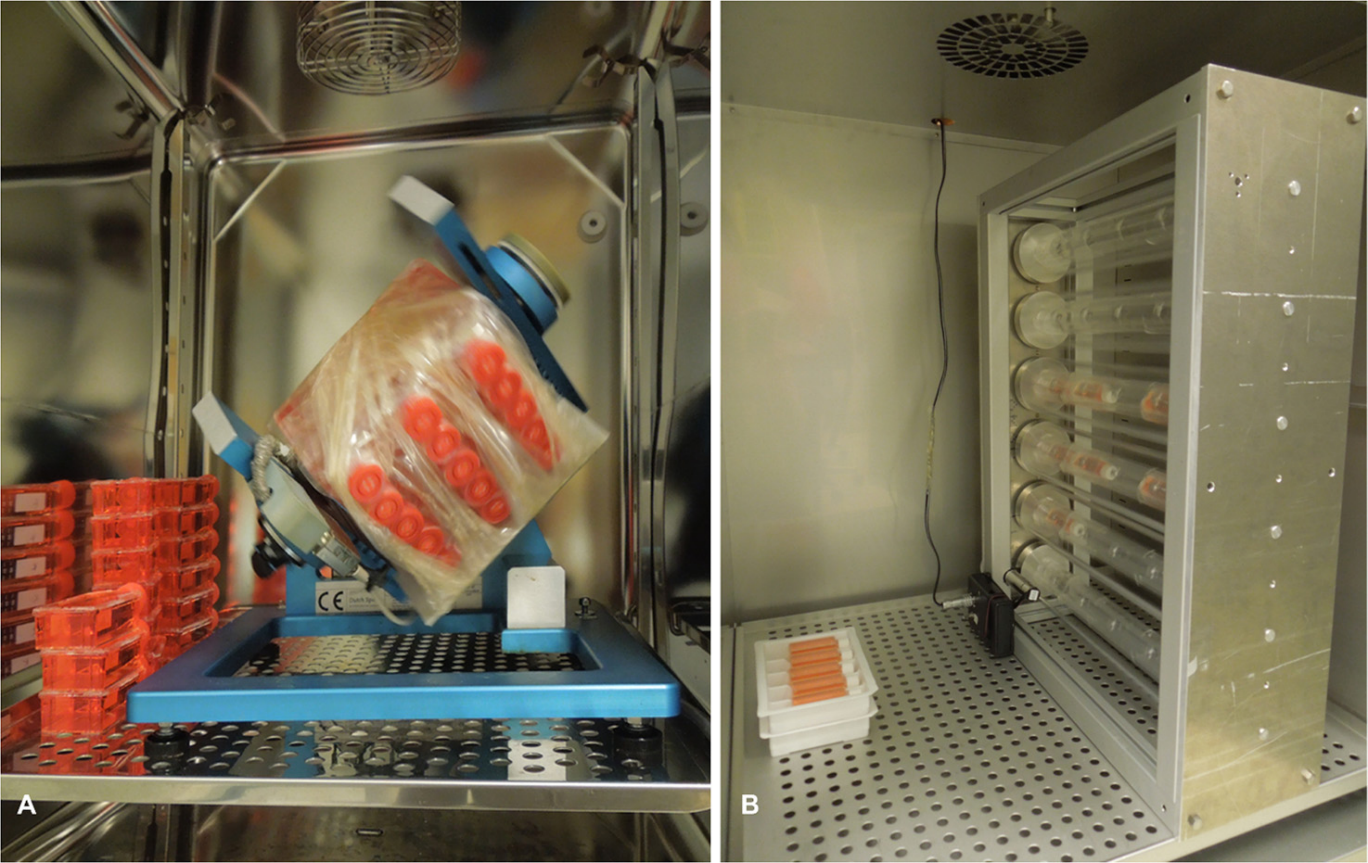
**A**: Random Positioning Machine (RPM) and **B**: 2D-Clinostat.

In recent years it became apparent that studies on the behavior of cancer cells in space might support cancer research on Earth [15]. Now it is of interest to compare the roles of distinct proteins in cellular adaption to changed environmental conditions (microgravity). We characterized various lines of human thyroid cancer cells grown under conditions of real and simulated microgravity with the aim to find possibilities of reducing the cancer cell aggressiveness [16–18]. Since experiments under real microgravity i.e. spaceflight possibilities are rare and expensive [16], a great part of the studies was performed using devices aiming to simulate microgravity on Earth [3, 19]. However, each device affects the cells not only by preventing sedimentation, but also by characteristics of its operation mode, which include transient hypergravity or vibration [20]. Therefore, it was considered that some observations made on cells cultured on a microgravity simulating device may not solely be due to preventing cell sedimentation but also due to device-specific effects [18]. Furthermore, we also observed that effects are specific for defined types of the thyroid cell lines [21]. In order to investigate the influence of altered gravity on the cellular level, we studied different cancer cells on different devices simulating microgravity according to comparable protocols. Prior to characterization, human thyroid cells FTC-133, ML-1, and HTU-5 were cultured on the Random Positioning Machine (RPM, Fig 1A) [17], but only FTC-133 cells on the RPM and the fast rotating 2D-Clinostat (CLINO, Fig 1B) [18] and in Space [16, 22, 23]. The experiments revealed several aspects and pointed to cytoskeletal proteins and cytokines as prime targets of microgravity effects [3, 19, 22, 23].

In this study we investigated the impact of simulated microgravity using the RPM and the CLINO devices on two human follicular thyroid cancer cell lines (ML-1, RO82-W-1) in a parallel manner either for three (3d) or seven (7d) days, respectively, before selected cytokines and cytoskeletal proteins were quantified. To evaluate the possible role of the cytokines IL-6 and IL-8 for the expression of selected proteins in thyroid cancer cells, we studied the impact of IL-6 and IL-8 application on Ki-67, ß_1_-integrin, talin-1, and beta-actin proteins in adherent ML-1 cells. Moreover, we focused on the role of the cytokines IL-6 and IL-8 in ML-1 and RO82-W-1 spheroid formation using the liquid-overlay technique under 1*g*-conditions [24]. Cytokines and cytoskeletal proteins, whose release or expression was altered, respectively, are discussed with respect to their specific roles in thyroid cancer.

## Methods

### Cell lines

#### ML-1 cell line

Monolayers of ML-1 follicular thyroid cancer cells [25] were cultured on either the RPM or the clinostat. The ML-1 cell line derived from a recurring tumor of a poorly differentiated follicular thyroid carcinoma (stage pT4) of a 50-year-old woman [25]. The cell line has a doubling time of 4 days. The cells take up glucose, secrete thyroglobulin, thyroxine, and triiodothyronine and are tumorigenic in nude mice.

#### UCLA RO82-W-1 cell line

The RO82-W-1 cell line was established by Estour *et al*. in 1989 [26]. The cell line was purchased from Sigma-Aldrich Chemie (Munich, Germany). This cell line derived from the metastases of a follicular carcinoma in a female patient. Although the primary tumor of RO82-W-1 released thyroglobulin (Tg) into the circulation, the uptake of I131 was less than 2% [26]. RO82-W-1 cells are Tg-positive and are also tumorigenic in nude mice [26].

Both cell lines were cultivated in RPMI-1640 medium at 37°C and 5% CO_2_. The medium was supplemented with 100 μg/mL streptomycin, 100 U/mL penicillin and 10% FCS (all Biochrom, Berlin, Germany).

Because both malignant human thyroid follicular cell lines had retained the ability to synthesize Tg, they represent valuable models for the study of human follicular carcinomas.

## Cell Culture Procedure

**Fig 2:**
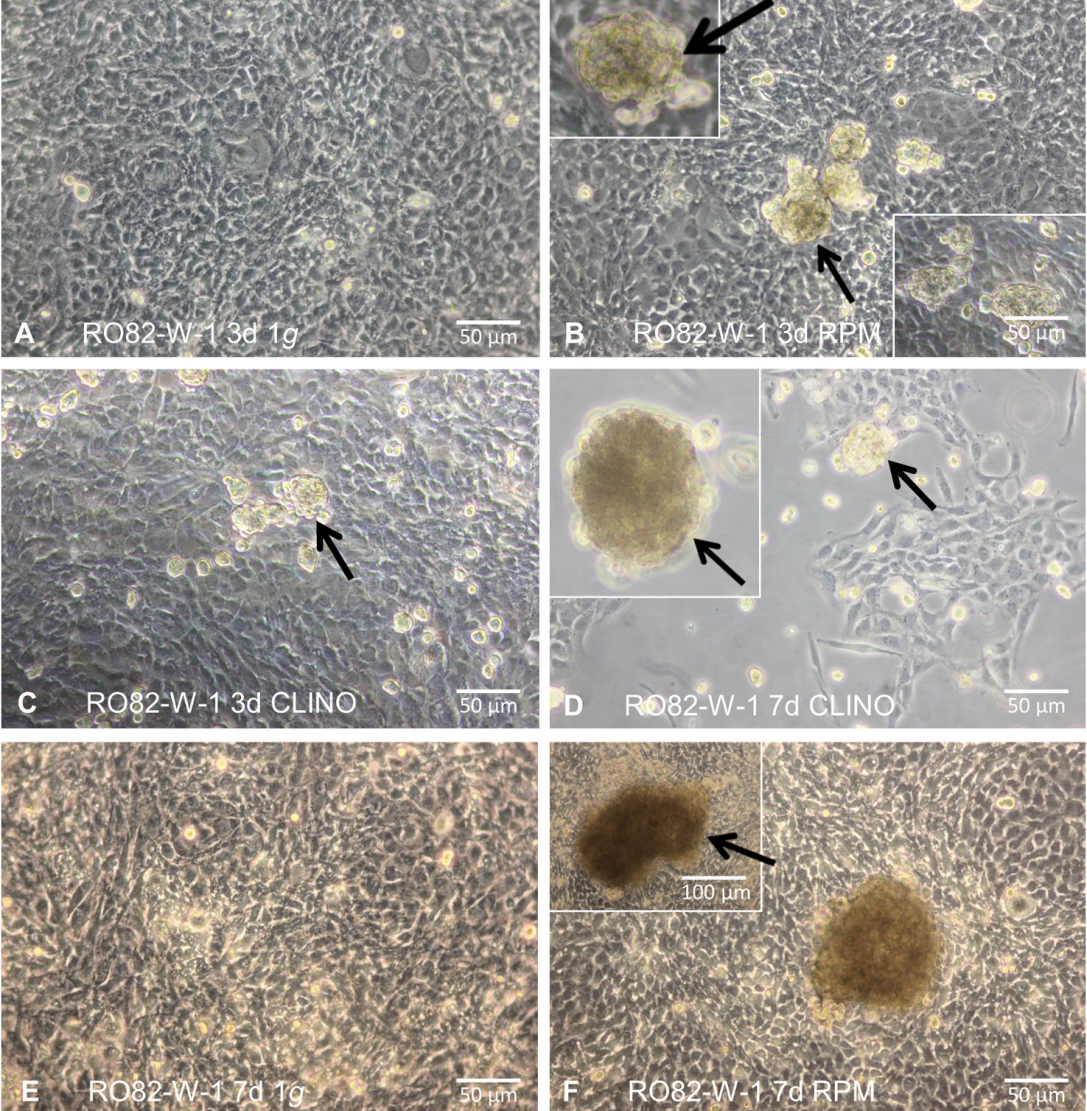
Phase contrast microscopy of RO82-W-1 cells. **A:** Follicular thyroid cancer cells cultured for 3d at static 1*g*. The cells proliferated as 2D monolayer. **B:** RO82-W-1 cells incubated for 3d on the RPM. The arrows show 3D aggregates (multicellular spheroids; MCS). The inserts show swimming 3D spheroids in the supernatant. **C:** MCS formed on the CLINO after 3 days. **D:** RO82-W-1 cells cultured for 7 days on the CLINO. The arrows show 3D spheroids. The insert demonstrates a floating spheroid in the supernatant. **E:** RO82-W-1 cells cultured for 7 days at static 1*g*-conditions grow as well as a confluent monolayer. **F:** 7-day-old MCS formed on the RPM (arrows) could be detected and adherent RO82-W-1 cells.

24 hours before the experiments, cells of both types were seeded into either slide flasks (Thermo Scientific, Roskilde, Denmark) with a growing area of 9 cm^2^ for the CLINO or into T25 flasks (Sarstedt, Nümbrecht, Germany) with a growing area of 25 cm^2^ for the RPM. The cells for each type of culture flasks were randomized to be cultivated as static ground controls (1*g*-condition) or on one of the devices. Phase contrast images were captured. The morphological pictures for RO82-W-1 cells are given in Fig 2. CLINO and RPM experiments were performed in individual incubators at 37°C, and ground controls were always kept next to the experiments in the same respective incubator, resting under static 1*g*-conditions. Cells of both types were harvested after 3d days and 7d.

### Effects of IL-6 and IL-8 on adherent ML-1 cells grown under 1*g*- conditions

ML-1 cells from frozen stocks were cultivated in T75 flasks in RPMI 1640 medium until they reached subconfluence (3–5 days). Afterwards the cells were subcultured in 5 T175 and as soon as they reached subconfluence they were subcultured again in 20 T175 flasks. After getting subconfluent, the cells were treated with vehicle RPMI 1640 medium or with 0.03 ng/mL, 1 ng/mL, 10 ng/mL, 100 ng/mL of IL-6 or 1 ng/mL, 10 ng/mL, 45 ng/mL or 100 ng/mL of IL-8 concentrations, respectively [27–29]. We also applied for the IL-6 group 0.03 ng/mL and for IL-8 45 ng/mL, because these concentrations were released in the supernatant by ML-1 cells after 7 days.

10 T175 flasks (2 T175 without IL-6 or IL-8 and 8 T175 with different concentrations of IL-6 or IL-8) were cultivated in the incubator (1*g*) for 3d, and another set of 10 T175 for 7d.

**Fig 3:**
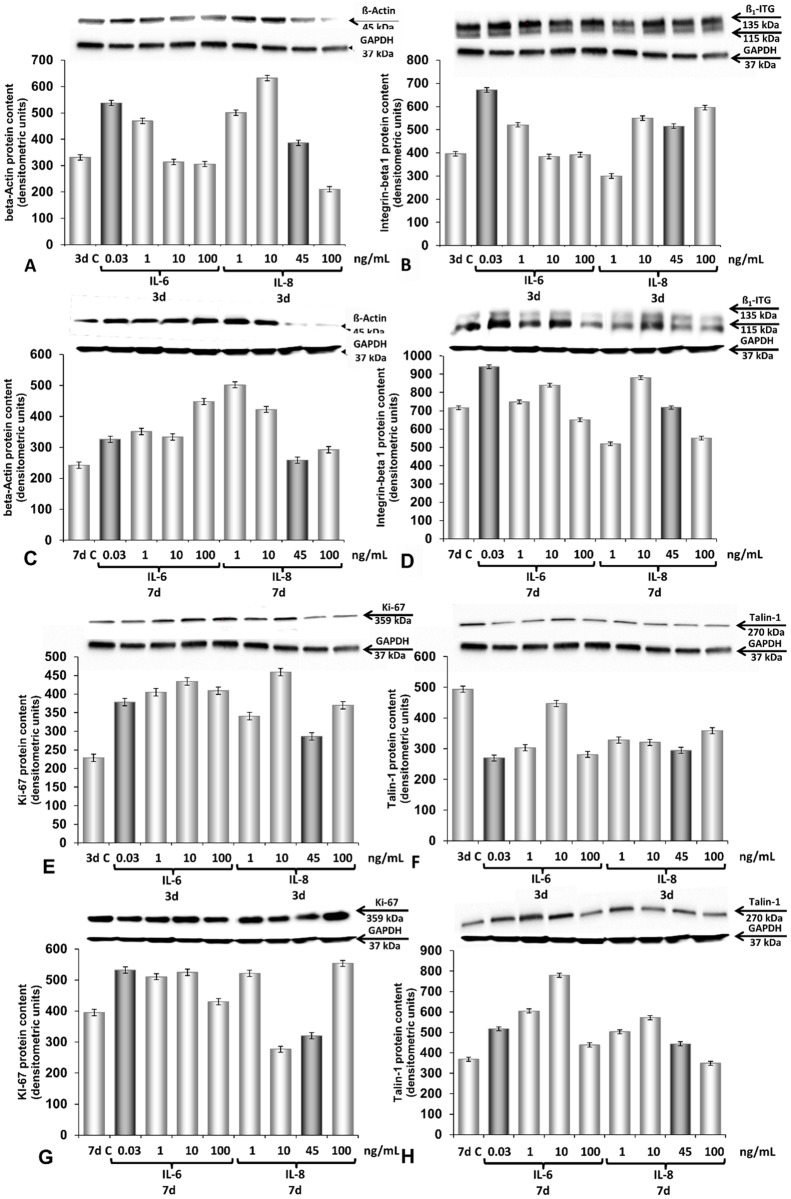
Effects of IL-6 and IL-8 on the amount of selected proteins in adherent ML-1 cells grown under normal 1*g*-conditions. Western blot analyses and densitometric data are given. **A, C:** beta-actin, 3d and 7d; **B, D:** ß_1_-integrin, 3d and 7d; **E, G:** Ki-67, 3 and 7 days; **F, H**: Talin-1, 3 and 7 days. IL-6 doses: 0.03 ng/mL; 1 ng/mL; 10 ng/mL and 100 ng/mL. The dose 0.03 ng/mL is the maximal amount of IL-6 released by ML-1 cells in the supernatant and measured by MAP (dark-grey columns). IL-8 doses: 1 ng/mL; 10 ng/mL; 45 ng/mL and 100 ng/mL. The dose 45 ng/mL is the maximal amount of IL-8 released by ML-1 cells in the supernatant and measured by MAP (dark-grey columns).

After 3d or 7d the medium in the culture flasks has been discarded, the cells were scraped in 10 ml of DPBS and centrifuged by 6000 rpm for 15 minutes. The supernatant was removed, the pellet was dissolved in 1 ml of DPBS and centrifuged again by 6000 rpm for 15 minutes. The pellets were immediately used for protein extraction, Western blot analysis of beta-actin, ß_1_-integrin, talin-1 and Ki-67 and following densitometry [17,19]. The results are given in Fig 3.

### Liquid-overlay technique

**Fig 4:**
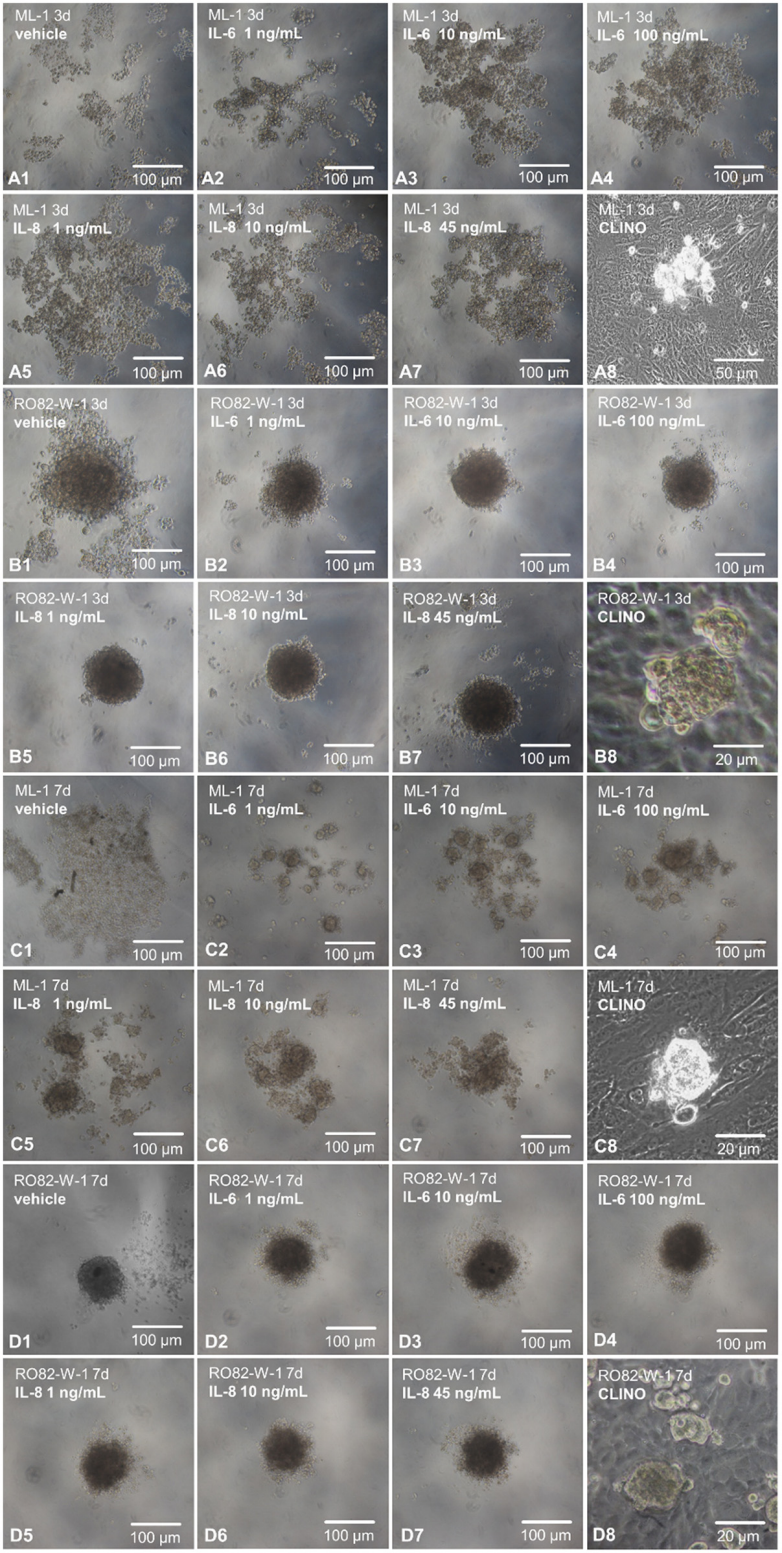
Liquid-overlay technique. **A1-A7:** Phase contrast microscopic pictures taken after 3d. ML-1 cells grown in agarose-coated wells, treated with vehicle or different doses of IL-6 or IL-8, respectively. **A8:** 3-day-old spheroid and adherent ML-1 cells on the CLINO. **B1-B7:** Photos taken after 3d. RO82-W-1 thyroid cancer cells grown in agarose-coated wells, treated with vehicle or different doses of IL-6 or IL-8, respectively. **B8:** 3-day-old spheroid and adherent RO82-W-1 cells on the CLINO. **C1-C7:** Pictures taken after 7d. ML-1 cells grown in agarose-coated wells, treated with vehicle or different doses of IL-6 or IL-8, respectively. B8: 7-day-old spheroid and adherent ML-1 cell population after incubation in the 2D CLINO. **D1-D7:** Phase contrast images taken after 7d. RO82-W-1 thyroid cancer cells grown in agarose-coated wells, treated with vehicle or different doses of IL-6 or IL-8, respectively. **D8:** 7-day-old MCS and adherent RO82-W-1 cells after culture in the CLINO.

Multicellular tumor spheroids were produced with the help of the liquid-overlay technique [24]. Thyroid cancer cells of the ML-1 cell line and the UCLA RO82-W-1 cell line harvested from adherently growing confluent monolayers were plated on agarose-coated 96-multiwell test plates (Nunc GmbH, Wiesbaden, Germany) at a concentration of 4000 cells/200 μL RPMI 1640 medium and were incubated at 37°C and 5% CO_2_. The cells were stimulated with vehicle, IL-6 (1 ng/mL, 10 ng/mL and 100 ng/mL) and IL-8 (1 ng/mL, 10 ng/mL and 45 ng/mL). After 3d and 7d pictures were taken and the results presented in Fig 4.

### Random Positioning Machine

The RPM was purchased from ADS, former Dutch Space, Leyden, the Netherlands (Fig 1A). Its construction and function was described earlier by van Loon [30]. It consists of two independent rotating frames within each other. In our experiments, the RPM was operated in “random mode” (60°/s), in which direction is changed continuously and randomly. Over time, the gravity vector of the Earth is nullified. During experiments, the RPM was loaded with up to 15 T25 cm^2^ flasks that were fixed to the ground plate of the machine. In effect, all flasks resided within a distance of 7.5 cm from the center of rotation. In the relatively low speed (60°/s) of the RPM, the centrifugal force experienced by cells even at the furthest points from the center of rotation is assumed to be negligible [30].

### 2D Clinostat

The 2D CLINO device (manufactured by DLR, Cologne, Germany, Fig 1B) consists of six arms enabling the rotation of the samples around an axis perpendicular to the force of gravity [31]. Each rotating arm was loaded with 4 slide flasks, for a total of 24 flasks per run. On the CLINO, the gravity vector is randomized by fast and constant rotation. Great care was taken to ensure that all flasks selected for clinorotation were free of air bubbles that would otherwise induce chaotic fluid movement. The 2D CLINO rotating constantly at 60 rpm generates residual accelerations within the distance of 3 mm around the center of rotation in the order of 0.012*g*, while cells located at further distance experience up to 0.036*g* [18, 31]. Although the gravity-related threshold of thyroid cancer cells is unknown, only the cells located within the distance of 3 mm around the rotational axis were harvested for the analyses, meaning that these cells had experienced a very low residual acceleration.

### pH measurements

The pH was measured with a Metrohm 827 pH-meter no more than 1 hour after experiment termination. All measurements were performed twice, and the samples were kept in closed Eppendorf tubes until measurement to avoid reactions with atmospheric gases.

### Phase contrast microscopy

The Axiovert 25 Microscope (Carl Zeiss Microscopy, LLC, USA) was used for visual observation of the morphology of the cells.

### Western blot analyses

Western blot analyses, immunoblotting, and densitometry were performed according to routine protocols [32–37]. The following antibodies were used to quantify the antigens: Anti-beta-actin, and anti-talin-1 were used at a dilution of 1:1000 (Cell Signaling Technology, Inc., Danvers, MA, USA); as well as anti-integrin-beta_1_ antibody (Epitomics, Burlingame, USA); Ki-67 was purchased from Santa Cruz Biotechnology, Santa Cruz, TX, USA (dilution 1:500); the secondary, HRP-linked antibody was utilized at a dilution of 1:4000 (Cell Signaling Technology, Inc., Danvers, MA, USA). As a loading control glyceraldehyde 3-phosphate dehydrogenase (ABR-Affinity BioReagents, Golden, USA; dilution: 1:10 000) was used.

The membranes were analyzed using ImageJ software (U.S. National Institutes of Health, Bethesda, MD, USA; http://rsb.info.nih.gov/ij/), for densitrometric quantification of the bands.

### F-actin staining

F-actin was visualized by rhodamine-phalloidin staining (Molecular Probes, Eugene, OR, USA) and the nuclei were stained with Hoechst 33342 (Molecular Probes, Eugene, OR, USA), as published earlier [36, 37]. The samples were mounted with Vectashield (Vector, Burlingame, CA, USA) and analyzed microscopically. The F-actin stained samples were examined using a Zeiss 510 META inverted confocal laser scanning microscope (Zeiss, Germany), equipped with a Plan-Apochromat 63×1.4 objective. Excitation and emission wavelengths were: λexc = 488 nm and λem = ≥505 nm for FITC. Afterwards, the samples were analyzed with the help of the image analysis program Scion Image (Version 1.63 MacOs, Scion Corporation, USA).

### Cytokine measurements by Multi-Analyte Profiling technology

The release of cytokines was investigated via Multi-Analyte Profiling (MAP) as previously described [18, 22]. For each condition, five supernatants were collected after 72 h and stored at -80°C until testing. The MAP was carried out by the company Myriad RBM (Austin, Texas, USA), they determined the Human Cytokines of the selections MAP A and B.

### Cytokine measurement by Enzyme Linked Immunosorbent Assay

IL-6, IL-8, and MCP-1 proteins released from RO82-W-1 cells in the cell culture supernatants during RPM and CLINO experiments were detected by ELISAs purchased from R&D systems [38]. The ELISAs have been performed according to the protocols supplied by the manufacturer.

### Statistical Evaluation

SPSS 15.0 (SPSS, Inc., Chicago, IL, USA) was used for statistical evaluation. The Mann-Whitney-U-Test was performed to compare 1 g and s-μg conditions, as well as s-μg adherent cells and s-μg MCTS cells. All data is presented as mean ± standard deviation (SD). Values of p < 0.05 were considered significant.

## Results

The cells of the follicular thyroid cancer cell lines ML-1 and RO82-W-1 were cultivated for 3d and 7d on the RPM, on the 2D CLINO, and under normal static laboratory 1*g*-conditions. Up to the seventh day, the pH remained in the normal range, irrespective of whether the cells were cultured on the RPM, under 1*g* or on the CLINO. However, the cells remained growing in a monolayer only if culturing was performed under 1*g*, while, when cultured under altered gravity conditions, the cells split into two populations of which one comprised cells remaining adherently, while the other contained cells forming 3D aggregates similar to those described for the FTC-133 cells [18].

We received similar results with the RO82-W-1 and ML-1 cells. Both types of cells proliferated like the RO82-W-1 cells shown in Fig 2 as an adherent monolayer (Fig 2A and 2E) and as multicellular spheroids on the RPM (Fig 2B and 2F) and the CLINO (Fig 2C and 2D). There were no differences in terms of number of spheroids of each cell line that arose on either the RPM or the CLINO. Both devices delivered spheroids of different sizes (max. diameter 0.4 mm) at both time points as shown in Fig 2B, 2C, 2D and 2F. Detachment of AD cells started early and cell detachment and spheroid formation occurred also after 7d.

### Impact of IL-6 and IL-8 on ML-1 cells grown under 1*g*-conditions

IL-6 (0.03 ng/mL and 1 ng/mL) stimulation increased the amount of beta-actin and ß_1_-integrin protein in adherent ML-1 cells. Moreover, IL-8 (1, 10 and 45 ng/mL) increased the beta-actin protein content in these cells, whereas only higher doses (10–100 ng/mL) elevated the protein content of ß_1_-integrin within 3 days (Fig 3A and 3B).

After 7d, an increase was found for beta-actin protein in all IL-6-treated samples as well as in samples treated with 1 and 10 ng/mL IL-8 (Fig 3C). The ß_1_-integrin protein was induced by 0.03 ng/mL as well as by 10 ng/mL IL-6, whereas only 10 ng/mL IL-8 induced the amount of the protein after a 7-day-stimulation (Fig 3D).

In addition, both cytokines (IL-6 and IL-8, all doses) induced an elevation of the Ki-67 protein content after 3d (Fig 3E). After 7d, all IL-6 doses as well as 1 ng/mL and 100 ng/mL IL-8 increased the amount of Ki-67 protein in ML-1 cells (Fig 3G). In contrast, the talin-1 protein content was clearly reduced by IL-6 and IL-8 application to the medium. Low doses of IL-6 and all doses of IL-8 were effective (Fig 3F) during the 3-day-1*g*-experiment. Another result was found after 7d. IL-6 stimulation resulted in an increase of talin-1 (Fig 3H). A similar finding was seen in IL-8-treated samples (1, 10 and 45 ng/mL) of the ML-1 cell line.

These experiments defined firstly, the optimal IL-6 and IL-8 doses for the expression of distinct proteins under 1*g*-conditions and secondly, the doses to test their impact on MCS formation using the liquid-overlay method (see below and Fig 4).

### Impact of IL-6 and IL-8 on spheroid formation under 1*g*-conditions

After 3d, ML-1 cells only showed loose cell aggregates, when cultured under 1*g*-conditions on agarose. 10 and 100 ng/mL IL-6 and 1, 10 and 45 ng/mL IL-8 treatment resulted in a better aggregation of the ML-1 cells to spheroids, when they are cultured in the agarose-coated wells (Liquid-overlay technique) (Fig 4A1–4A7). RO82-W-1 cells formed large irregular aggregates after a 3-day-incubation in agarose-coated wells. Both cytokines improved the formation of RO82-W-1 multicellular spheroids using the liquid-overlay method (Fig 4B1–4B7).

After 7d, ML-1 cells showed irregular formed cellular aggregates on agarose. The cytokine-treated groups contained several dense spheroids after one week of stimulation (Fig 4C). The ML-1 spheroids formed on the CLINO were similar to the liquid-overlay spheroids treated with 45 ng/mL IL-8 (Fig 4C7 and 4C8).

After 7d, the RO82-W-1 cells grew in form of dense multicellular spheroids as well as single cells swimming in the supernatant in agarose-coated wells. Cytokine application to the medium enhanced slightly the size of the spheroids (Fig 4D1–4D7).

The spheroids of both cell types formed after IL-6 and IL-8 treatment exhibited a similar morphology than the MCS produced after incubation on the CLINO (Fig 4A8, 4B8, 4C8 and 4D8).

### Cytokine release by ML-1 cells

**Fig 5:**
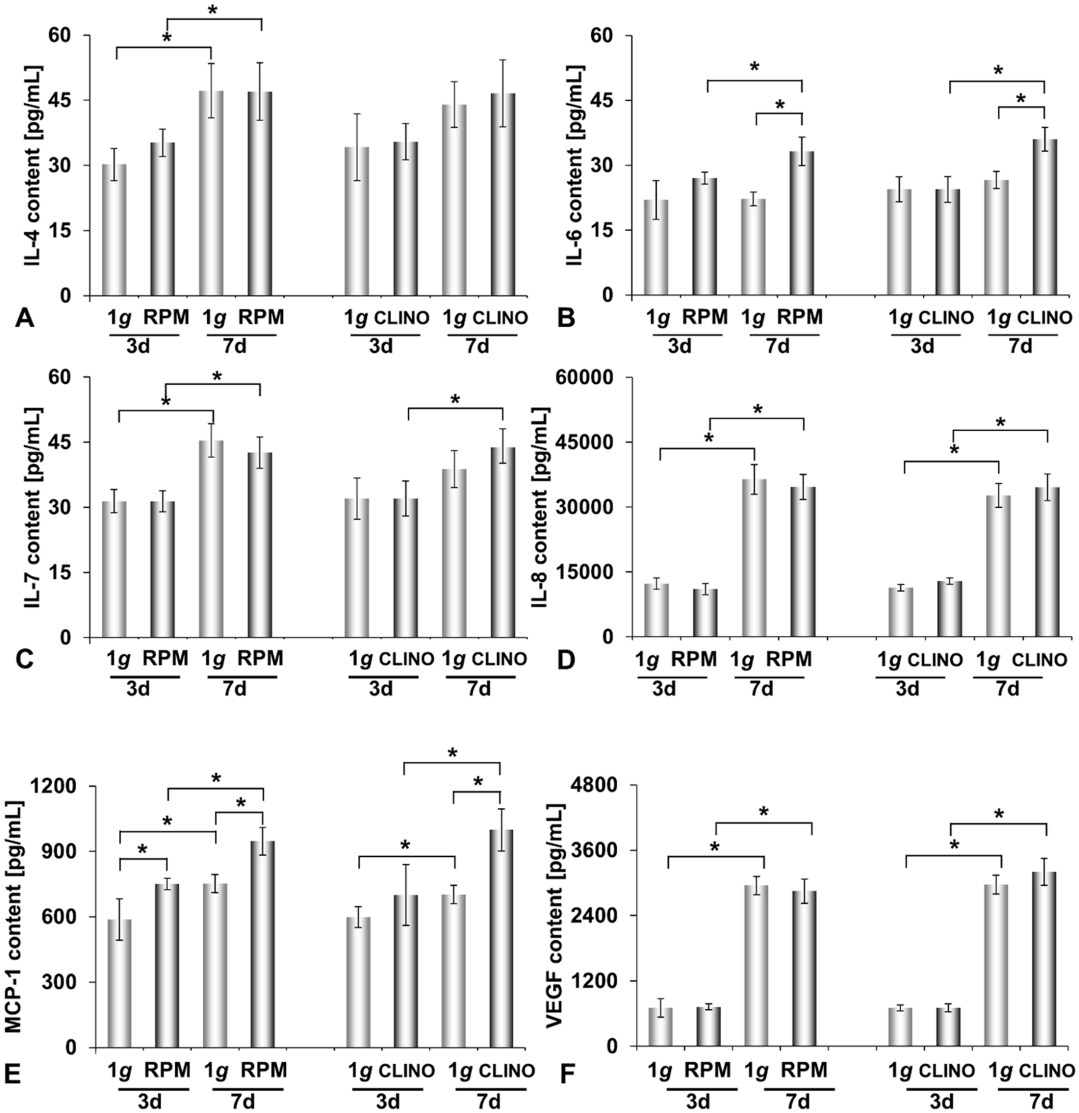
**A**: IL-4, **B**: IL-6, **C**: IL-7, **D**: IL-8, **E**: MCP-1, **F**: VEGF-A proteins released by ML-1 cells into the supernatant after a 3- and 7-day-exposure to the RPM or the 2D Clinostat, determined by Multi-Analyte Profiling of the supernatant. RPM: Random Positioning Machine; CLINO: 2D Clinostat; *p<0.05

Measuring the cytokines of the Human Cytokine selections MAP A and B we detected IL-4, IL-6, IL-7, IL-8, IL-17, MCP-1, VEGF-A and eotaxin-1 in the supernatants of the various cell cultures. We measured about 32 pg/mL IL-4 and IL-7 in the supernatants of 3-day-old cell cultures, irrespective of whether the cells had been incubated on the RPM, on the CLINO or in the stationary 1*g*-control. After a 7-day-exposure, the cell culture supernatants contained around 47 pg/mL IL-4 and about 43 pg/mL Il-7, independently of the culture conditions (Fig 5A and 5C). A similar phenomenon was observed with respect to IL-8 and VEGF-A. About 710 pg/mL VEGF-A and 11,000 pg/mL IL-8 were found in the supernatants of 3-day-old cultures, irrespective of whether the cells had been incubated on the RPM, on the CLINO or under stationary 1*g*-conditions. After a 7-day-exposure, VEGF-A and IL-8 concentrations were enhanced to around 3000 pg/mL VEGF-A and about 35,000 pg/mL Il-8 were detected, again without significant difference with respect to culturing conditions (Fig 5D and 5F). Taken together, Fig 5A, 5C, 5D and 5F show that the content of IL-4, IL-7, VEGF-A and IL-8 in the culture supernatants were enhanced in the 7-day-samples as compared to the 3-day-samples, while a significant influence of exposing cells to the RPM or the CLINO could not be seen for these 4 types of cytokines.

In contrast, the accumulation of IL-6 and MCP-1 in the different culture supernatants clearly depended on the culture conditions. A release of 27 and 33 pg/mL IL-6 was found in the supernatants of RPM samples after 3d and 7d of culturing, respectively, while an amount of 22.5 pg/mL IL-6 was measured in relevant 1*g*-controls (Fig 5B). Similarly, a concentration of 24 and 36 pg/mL IL-6 was found in the supernatants of CLINO samples after a 3- and 7-day-experiment, respectively, while about an amount of 25 pg/mL IL-6 was measured in relevant 1*g*-controls (Fig 5B). In addition, the monocyte chemoattractant protein-1 (MCP-1) levels changed, when cell culturing was performed on devices simulating microgravity. Under normal gravity conditions, about 600 pg/mL MCP-1 were found after 3 days and between 700 and 750 pg/mL after 7 days, while MCP-1 concentrations increased from 750 to 950 pg/mL on the RPM and from 700 to 1000 pg/mL on the CLINO (Fig 5E).

In addition to the six cytokines shown in Fig 5 we searched for further cytokines as shown in Table 1. However, we did not detect further cytokines released into the supernatant during 3 days of cell culturing under any condition. Release of IL-17 and eotaxin was detectable in samples cultured for 7 days on the CLINO, on the RPM as well as on the ground with no significant differences at concentrations of around 1 pg/mL (IL-17) and 15 pg/mL (eotaxin) (Table 1).

**Table 1:** Soluble factors secreted by ML-1 cells.

	Factor	LDD		Random Positioning Machine				Clinorotation			
				3d	3d control	7d	7d control	3d	3d control	7d	7d control
**Human CytokineMAP A 1.0**	**GM-CSF**	2,9	[pg/ml]	n.d.	n.d.	n.d.	n.d.	n.d.	n.d.	n.d.	n.d.
	**IFN-γ**	0,32	[pg/ml]	n.d.	n.d.	n.d.	n.d.	n.d.	n.d.	n.d.	n.d.
	**IL-2**	1,2	[pg/ml]	n.d.	n.d.	n.d.	n.d.	n.d.	n.d.	n.d.	n.d.
	**IL-3**	0,00032	[pg/ml]	n.d.	n.d.	n.d.	n.d.	n.d.	n.d.	n.d.	n.d.
	**IL-5**	0,55	[pg/ml]	n.d.	n.d.	n.d.	n.d.	n.d.	n.d.	n.d.	n.d.
	**IL-10**	0,66	[pg/ml]	n.d.	n.d.	n.d.	n.d.	n.d.	n.d.	n.d.	n.d.
	**IL-18**	1,3	[pg/ml]	n.d.	n.d.	n.d.	n.d.	n.d.	n.d.	n.d.	n.d.
	**MIP-1α**	3,6	[pg/ml]	n.d.	n.d.	n.d.	n.d.	n.d.	n.d.	n.d.	n.d.
	**MIP-1β**	3,2	[pg/ml]	n.d.	n.d.	n.d.	n.d.	n.d.	n.d.	n.d.	n.d.
	**TNF-α**	2,6	[pg/ml]	n.d.	n.d.	n.d.	n.d.	n.d.	n.d.	n.d.	n.d.
	**TNF-β**	0,6	[pg/ml]	n.d.	n.d.	n.d.	n.d.	n.d.	n.d.	n.d.	n.d.
**Human CytokineMAP B 1.0**	**BDNF**	0,0054	[ng/ml]	n.d.	n.d.	n.d.	n.d.	n.d.	n.d.	n.d.	n.d.
	**Eotaxin-1**	13	[pg/ml]	n.d.	n.d.	13.4 ± 7.9	17.6 ± 2.3	n.d.	n.d.	16.4 ± 1.8	13.6 ± 7.8
	**ICAM-1**	0,65	[pg/ml]	n.d.	n.d.	n.d.	n.d.	n.d.	n.d.	n.d.	n.d.
	**IL-1α**	0,0003	[pg/ml]	n.d.	n.d.	n.d.	n.d.	n.d.	n.d.	n.d.	n.d.
	**IL-1β**	0,44	[pg/ml]	n.d.	n.d.	n.d.	n.d.	n.d.	n.d.	n.d.	n.d.
	**IL-1ra**	31	[pg/ml]	n.d.	n.d.	n.d.	n.d.	n.d.	n.d.	n.d.	n.d.
	**IL-12p40**	0,019	[ng/ml]	n.d.	n.d.	n.d.	n.d.	n.d.	n.d.	n.d.	n.d.
	**IL-12p70**	6,8	[pg/ml]	n.d.	n.d.	n.d.	n.d.	n.d.	n.d.	n.d.	n.d.
	**IL-15**	0,039	[ng/ml]	n.d.	n.d.	n.d.	n.d.	n.d.	n.d.	n.d.	n.d.
	**IL-17**	0,31	[pg/ml]	n.d.	n.d.	1.078 ± 0.229	1.2 ± 0,071	n.d.	n.d.	1.12 ± 0.16	1.02 ± 0.602
	**IL-23**	0,13	[ng/ml]	n.d.	n.d.	n.d.	n.d.	n.d.	n.d.	n.d.	n.d.
	**MMP-3**	0,0052	[ng/ml]	n.d.	n.d.	n.d.	n.d.	n.d.	n.d.	n.d.	n.d.
	**SCF**	13	[pg/ml]	n.d.	n.d.	n.d.	n.d.	n.d.	n.d.	n.d.	n.d.
										

Values are given with mean ± SD; 1*g*, corresponding ground control; n.d., not detectable; LDD (Least Detectable Dose)-determined as the mean ± 3 standard deviations of 20 blank readings.

### Cytokine release by RO82-W-1 cells

**Fig 6:**
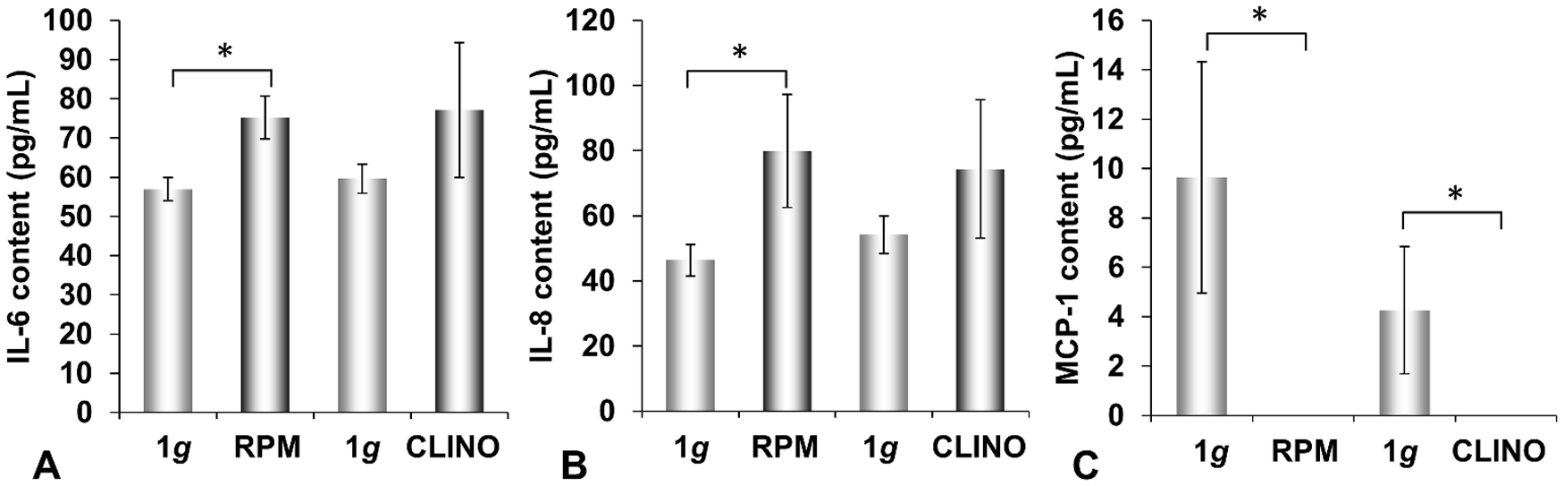
**A:** IL-6, **B:** IL-8, and **C:** MCP-1 proteins released by RO82-W-1 thyroid cancer cells into the supernatant after a 3-day-exposure to the RPM or the 2D CLINO, determined by ELISA technique. RPM: Random Positioning Machine; CLINO: 2D Clinostat; *p<0.05.

We investigated the release of selected cytokines in the supernatant of RO82-W-1 cells after a 3-day-exposure to the RPM or the CLINO devices. The amount of IL-6 was significantly elevated from 56.9 pg/mL to 75.2 pg/mL on the RPM (Fig 6A). A similar result was obtained for IL-8 (Fig 6B). Non-significant increases for both cytokines were measured for the CLINO-samples.

The secretion of MCP-1 of RO82-W-1 cells was much lower than the release of ML-1 cells (Figs 5E and 6C). Culture of RO82-W-1 cells on the RPM or the CLINO completely blunted the release of MCP-1 (Fig 6C).

### Effects of simulated microgravity on the cytoskeleton

**Fig 7:**
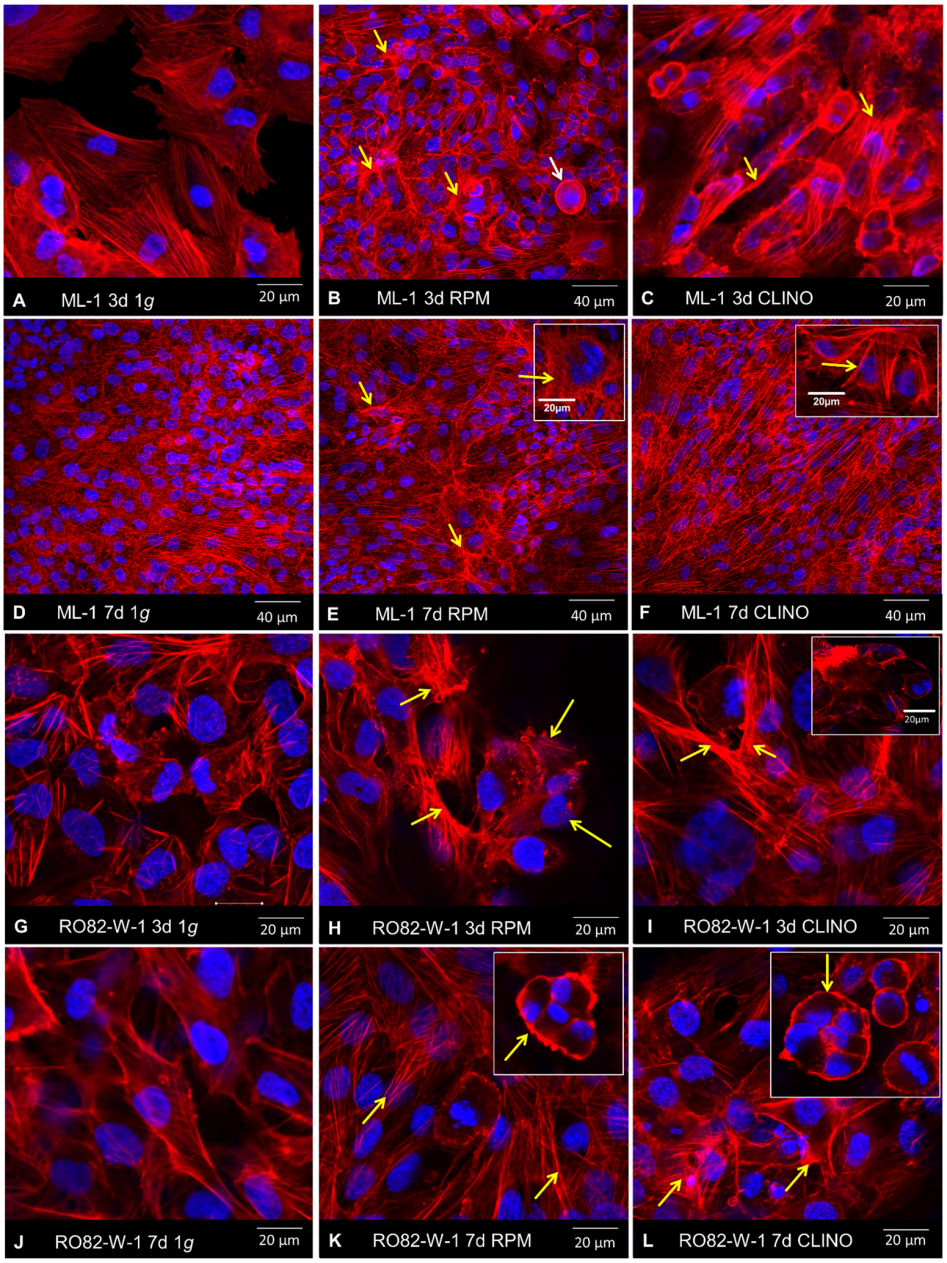
F-Actin staining of ML-1 and RO82-W-1 cells after 3d and 7d on the RPM and on the CLINO. **A-C:** ML-1 3-day-experiment. **A:** ML-1 cells, 3d, 1*g*-condition: normal cells with normal F-actin cytoskeleton **B:** ML-1 cells, 3d, accumulation of F-actin at the outer cellular membrane (yellow arrows) after RPM-exposure. The white arrow marks the detaching of cell aggregates from the adherent cell layer. **C:** ML-1 cells, 3d, thicker layers of F-actin at the outer cellular membrane (yellow arrows). **D-F:** ML-1 7-day-experiment. **D:** ML-1 cells, 7d, 1*g*-condition: confluent normal cells with normal F-actin cytoskeleton. **E:** ML-1 cells cultured on the RPM for 7d revealed thicker layers of F-actin at the outer membrane (insert). **F:** A 7-day-exposure of ML-1 cells to the CLINO induced similar changes of the F-actin cytoskeleton. The insert shows ticker F-actin fibers at the cellular membranes. **G-I:** RO82-W-1 3-day-experiment. **G:** normal F-actin cytoskeleton of RO82-W-1 cells. **H:** MCS formation (yellow arrows) after a 3-day-incubation on the RPM. An accumulation of F-actin at the outer cellular membrane is visible (yellow arrows). **I:** RO82-W-1 cultured for 3d on the clinostat revealed a clear thickening of the F-actin fibers (arrows). Insert: 3D MCS. **J-L**: RO82-W-1 7-day-experiment. **J:** F-actin cytoskeleton of confluent RO82-W-1 cells grown at 1*g*. **K, L:** MCS formation of RO82-W-1 cells after RPM- (**K**) and CLINO-exposure (**L**). The arrows indicate the 3D MCS and a thickening of F-actin fibers at the outer membranes.

Since earlier studies have shown that cytoskeletal proteins of different types of cells are severely affected by microgravity [11–13, 37, 39–41], we performed F-actin staining on cells cultured on the RPM and on the 2D CLINO for 3d and 7d. After 3 days, an accumulation of F-actin along the outer cellular membrane was visible in ML-1 cells as well as in RO82-W-1 cells cultured on the CLINO and the RPM (Fig 7). After 7 days, the cells were completely confluent and overgrew each other. A thickening of the outer membrane was found under both conditions (1*g* and simulated microgravity), but was more pronounced on the RPM and the CLINO.

**Fig 8:**
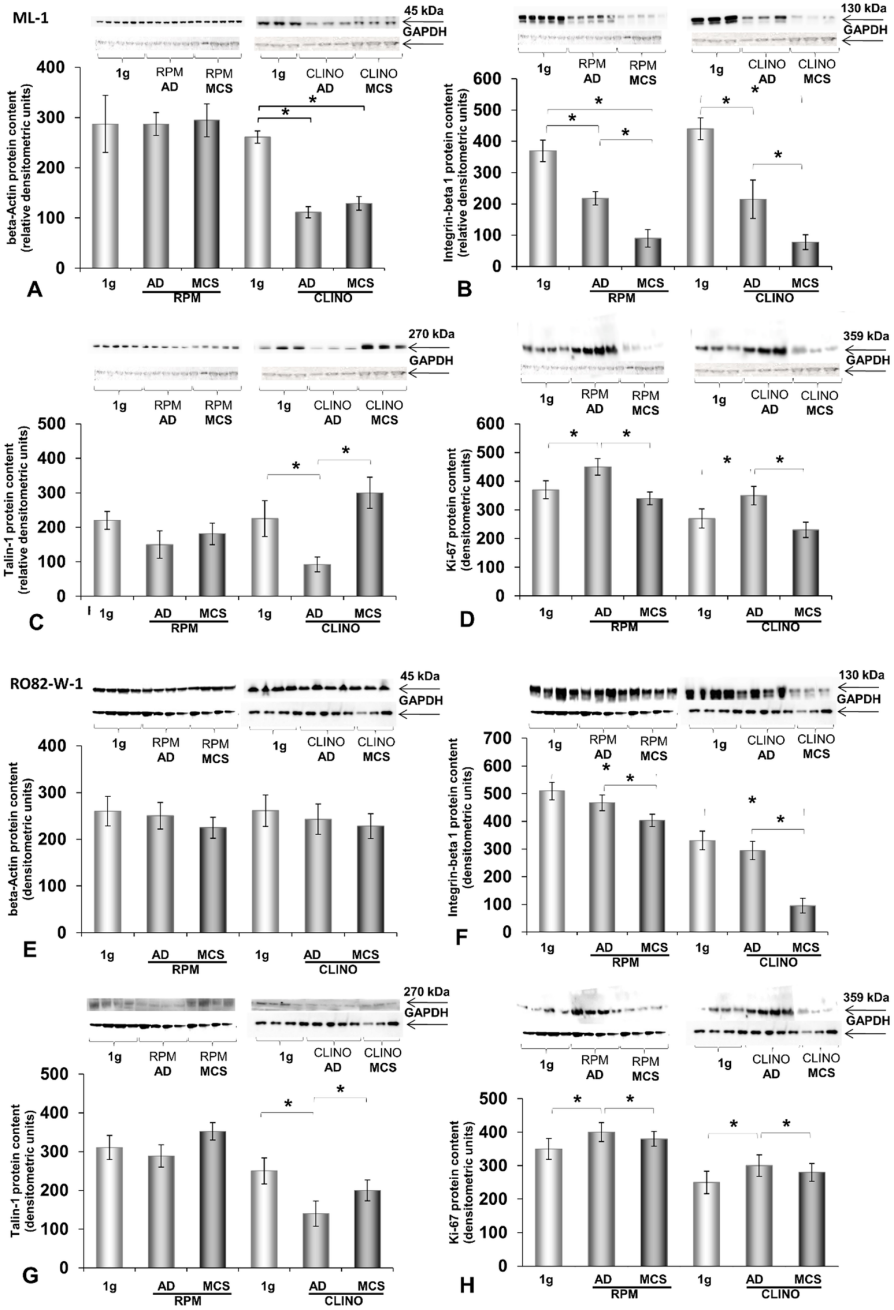
Western blot analyses of **A-D:** ML-1 cells after a 7-day-exposure on the RPM and CLINO. **A**: ß-actin, **B**: ß_1_-integrin, **C**: talin-1 and **D:** Ki-67 proteins. Clear differences between the two devices are found for ß-actin. Western blot analyses of **E-H:** RO82-W-1 cells after a 7-day-exposure on the RPM and CLINO. **E:** ß-actin, **F:** ß_1_-integrin, **G:** talin-1 and **H:** Ki-67 proteins. There was no change in the beta-actin content of RO82-W-1 cells cultured on the RPM or the CLINO for 7d. The amount of ß_1_-integrin and Ki-67 was comparable to the ML-1 cultures grown under s-μ*g*. *P<0.05.

Western blot analyses of β-actin in ML-1 cells revealed a decrease after 7d of clinorotation (Fig 8A), but the protein remained unchanged after 7d of RPM-exposure. Exposure of the ML-1 cells to the RPM and clinostat induced significant decreases of β_1_-integrin in AD and MCS cells after 7d (Fig 8B). Talin-1 protein was significantly reduced in AD cells only, when the ML-1 cells were incubated on the CLINO (Fig 8C). Ki-67 protein, a nuclear protein, associated with the cellular proliferation process, was significantly elevated in AD cells compared with MCS and 1*g*-control cells on both devices (Fig 8D).

No change in beta-actin was found in RO82-W-1 cells after a 7-day-culture on the RPM or the CLINO (Fig 8E). The protein content of beta-actin remained constant. The protein contents of beta_1_-integrin, talin-1 and Ki-67 of RO82-W-1 cells were comparable to those of the ML-1 cell line, irrespective of culture conditions (Fig 8F–8H).

## Discussion

Ground-based facilities such as the RPM and the CLINO, used to simulate conditions of microgravity on Earth, have various benefits. They are important tools to prepare a future space mission [3, 4, 7, 30, 39–42]. They allow us also to explore altered gravity (microgravity)–related effects on Earth. However, most often they show superimposing effects, which may be due to the characteristics of a device. Common effects exerted by the RPM and the CLINO on target cells of interest may be induced due to prevention of sedimentations. We have recently demonstrated that the CLINO and the RPM are suitable to trigger FTC-133 cells to develop MCS and to decrease expression of CAV1 and CTGF genes in MCS [18]. In this study we focused on the regulation of cytokines and cytoskeletal proteins of two follicular thyroid cancer cell lines ML-1 and RO82-W-1 under the influence of the devices. In Ml-1 cells, we observed an enhanced release of IL-6 and MCP-1, an elevated Ki-67 protein content in AD cells, but a reduced production of ß_1_-integrin and talin-1 in AD cells. RO82-W-1 cells exhibited a similar production of these proteins, when the cells were exposed to simulated microgravity conditions. The release of IL-6 and IL-8 was enhanced in RO82-W-1 cells, but MCP-1 secretion was blunted under conditions of microgravity. Different reactions of the two cell lines under CLINO- or RPM-exposure might be due to their different origin and characteristics.

Since both machines trigger similar cytoskeletal changes in both cell lines, we concluded that these alterations might be important in adaptation to microgravity [4, 11–13, 36, 37]. Furthermore, the proteins investigated play also a role in cancer development and progression.

A number of cytokines are known to play a role in tumor cell proliferation, apoptosis, and angiogenesis [43]. IL-4 has been described as a growth factor of thyroid cancer cells. It promotes resistance to chemotherapy by upregulating secondary messengers [44]. The role of IL-7 in relation to thyroid cancer remains largely unknown. Increased serum levels of IL-7 in conjunction with IL-6, IL-10 and IL-14 had been shown to indicate both benign and malignant thyroid disease [45]. IL-8 plays a role in arthroid disease [46]. In addition, IL-8 is responsible to tumor progression and liver metastasis of colorectal tumors [47]. IL-17 plays a role in tumor development, but the mechanisms of IL17 involvement are still uncharacterized [48]. Also VEGF promotes neoangiogenesis. It is produced by many thyroid cancer cells and supports infiltration of thyroid tumors by vasculature and thus promotes tumor growth [49, 50]. Its neutralization by antibodies or drugs is currently of great interest in tumor therapy [51, 52]. All these cytokines were accumulated in supernatants of ML-1 cultures progressively during time, while a number of other cancer cell-related cytokines could not be detected in the various supernatants (Table 1). There was no gravity-dependent influence on the secretion of the various cytokines by ML-1 cells mentioned in Table 1 and of those described above besides slight differences found for IL-7 and IL-8 (Fig 5). Hence, we concluded that a number of cytokines, playing a role in tumor development are not affected by culturing the cells on the devices applied. It is of interest that also the amounts of VEGF-A and eotaxin in the supernatants of the FTC-133 cells cultured during the Shenzhou-8 flight in real microgravity and on 1*g*-reference centrifuge were equal, respectively [22].

The up-regulation of IL-6 and MCP-1 occurred in RPM and CLINO samples of the ML-1 cell line (Fig 5B and 5E). IL-6, along with IL-8, had already been suggested to play a role in gravity-sensitive signaling required for spheroid formation [19]. To investigate the role of IL-6 and IL-8 on spheroid formation under 1*g*-conditions, we used the liquid-overlay technique [24]. We could show for the first time that both cytokines improve 3D aggregation of both ML-1 and RO-82-W-1 cells, which were cultured on agarose-coated wells for 3d and 7d (Fig 4). The 3- and 7-day-old MCS were comparable to the spheroids grown under conditions of simulated microgravity. In addition, we demonstrated that both cytokines induced the protein expression of beta-actin, beta_1_-integrin, talin-1 and Ki-67 within 3 and 7 days (Fig 3). These results support recent research suggesting that IL-6 might be an important factor in tumor cell growth, metastasis and angiogenesis [53]. Immunohistochemical staining of papillary thyroid tumor samples indicated that MCP-1 expression correlated with an aggressive behavior of this tumor [54]. MCP-1 was highly secreted and elevated in ML-1 cells cultured under simulated microgravity. This cell line derived from a poorly differentiated follicular thyroid carcinoma. In contrast, RO82-W-1 cells exhibited a low secretion rate of this cytokine. A 3-day-incubation on the RPM and CLINO blunted the secretion of MCP-1 (Fig 6C). In space during the Shenzhou-8 mission, we also had measured after 10 days in FTC-133 supernatants 8pg/mL MCP-1 in spaceflight 1g-samples and 6.15 pg/mL MCP-1 in real μg-samples [22]. This reduction is in accordance with the results obtained from RO82-W1 samples, which also exhibited a reduction for this cytokine.

In addition, we investigated the F-actin cytoskeleton of the thyroid cancer cells. F-actin accumulated at the outer cellular membranes in both types of follicular thyroid cancer cells cultured on both devices for 3d and 7d (Fig 7), corresponding to our earlier studies on endothelial cells and human chondrocytes [4, 36, 37, 40] and further proves cytoskeletal alterations in cells cultured under microgravity conditions as reported by several authors [11–13, 55–57]. It is well known that microgravity induces changes in the actin cytoskeleton, which is connected to several membrane proteins with impact on cellular polarity, adhesion, migration, and may respond to extracellular signals [55]. These alterations occur very early. After the first microgravity phase of a DLR Parabolic Flight a clear rearrangement of the F-actin network with perinuclear clustering was demonstrated in ML-1 cells [55]. In addition, MCS formation occurred after 3d and 7d, a thick F-actin layer is visible at the outer membrane of the developing spheroids (Fig 7K and 7L).

Furthermore, we found a significant reduction of β-actin in CLINO-samples of the ML-1 cell line, whereas, a 7-day-RPM-exposure did not alter the expression of the protein (Fig 8A). Interestingly, RO82-W-1 cells exhibited no change in β-actin after a 7-day-culture on the RPM and the CLINO (Fig 8E). This protein is one of two non-muscular cytoskeletal actin types and is important for the stability of cells as well as for motility and structure. β-actin is expressed by tumor cells as well as by healthy cells, and overexpression of β-actin had been found to be associated to metastasis of gastric cancer [58], however, its role in thyroid cancer cells is currently unknown. The finding that β-actin is not changed in RO82-W-1 cells and the differences found for CLINO- and RPM-ML1 samples may be explained as follows: The notable differences between the amounts of β-actin on the RPM vs. the clinostat could be firstly, due to patient-specific properties of the two cell lines. Secondly, they may occur because of device characteristic reasons. The RPM consists of two independently rotating frames enabling a 3D rotation with random speed and random direction of the samples aiming to alter the influence of the gravity vector [30]. This might induce stress experienced by cells on the RPM when the direction is changed, where cells will experience numerous brakes for changing the vector and for inducing gravitational unloading may be also one possible reason for the difference. ML-1 cells may be more sensitive and can stabilize their cytoskeleton by producing more β-actin on the RPM [7, 30]. This does not occur in the CLINO due to constant rotating with respect to speed and direction. Moreover, on the CLINO, sedimentation is prevented by a fast and constant rotation of the samples around one horizontal axis, assuming that the sample does no longer perceive the gravity stimulus.

Both cell lines exhibit MCS formation on both devices. During MCS formation considerable alterations of various cellular molecules can occur. Some of them might not be directly related to the process of 3D cell aggregation, but by products generated by device-dependent modifications [59]. Examples are physical stimuli due to vibration, shearing forces, etc. generated by the simulators [18]. The CLINO device rotates constantly at 60 rpm and generates residual accelerations below 0.012*g* within the distance of ±3 mm around the culture flask center [31]. Cells located at further distance from the rotation axis are exposed to accelerations reaching up to 0.036 g at about ±9 mm [18]. Eiermann *et al*. had demonstrated significant differences in several genes in a human 1F6 melanoma cell line within these acceleration intervals [31]. To be sure to keep residual acceleration low, we only collected cells within the central 6 mm (≤ 0.012 g) of a slide flask were collected and analyzed by Western blot analysis in the present study. Such a procedure is not possible for supernatants, and therefore IL-6 and IL-8 as well as all other soluble factors produced by cells from the periphery have a significant influence on cells growing in the center of the slide flasks. This may be one reason for the differences in beta-actin and also talin-1 protein expression. Most importantly, research in simulated microgravity has to be confirmed by a real space experiment.

β_1_-integrin belongs to a family of adhesion molecules and is known to be important in binding the basal membrane and release proteases, thus facilitating the invasion of cancer cells into the surrounding tissue. Follicular as well as papillary thyroid carcinoma cells express increased levels of β_1_-integrin [60, 61]. Talin is a high-molecular weight cytoskeletal protein, which links integrins to the actin cytoskeleton [62]. Its overexpression enhanced prostate cancer cell adhesion, migration, and invasion [63]. β_1_-integrin and talin-1 are reduced in adherent ML-1 cells. MCS cells of both cell lines contain a lower amount of β_1_-integrin, whereas talin-1 is not significantly changed in MCS as compared with 1*g*-samples, irrespective of the used microgravity simulator (Fig 8). The protein content of talin-1 in RO82-W-1 MCS is higher than in AD cells on both devices. The differences between the RPM and CLINO devices as already discussed may be the reason for this finding. In an earlier study we had already investigated ML-1 cells for 72 h on a 3D clinostat and detected an increase in ß_1_-integrin in AD cells [64]. After 72 h the protein content of AD cells was comparable to the content of MCS samples, but remained elevated as compared to 1*g*-samples [64]. Cellular adhesion and adhesion to the extracellular matrix is very important for the organism and is mediated by integrins. This process occurs early, which might explain why after a 7-day-exposure to s-μg, ß_1_-integrin was reduced in MCS and AD cells. Talin is also required for a normal integrin function and is necessary for the activation of ß_1_-integrins in many cell types. When ML-1 cells were cultured under s-μg, talin was early elevated and MCS exhibited a higher amount of talin compared with 1*g* and AD cells [64]. Talin is a cytoskeletal actin-binding protein that binds to integrin and also colocalized with activated integrins. Therefore, an early increase of this protein in parallel to ß_1_-integrin can be expected in microgravity. Afterwards, the cells produce a constant level of this protein on the RPM.

Moreover, we evaluated the proliferation of the cells and measured the amount of Ki-67 protein. Ki-67 is an excellent proliferation marker, which is detectable during the cell cycle phases G1, S, G2, and mitosis, but is absent from resting cells (G0). It indicated a high proliferation in 7-day-old ML-1 and RO82-W-1-cultures, which was higher in AD than in MCS (Fig 8D and 8H). MCS exhibited an unaltered Ki-67 content compared with corresponding controls (Fig 8D and 8H). The Ki-67 content was also measured in human endothelial cells [41]. In human EAhy926 cells cultured on an RPM, equal amounts of Ki-67 were detectable in adherent cells under both 1*g-* and s-μg-conditions. Ki-67 was reduced by about 50% in MCS floating in the culture medium after 7 days under simulated microgravity [41].

In summary, these results support the hypothesis that simulated microgravity using the RPM or the CLINO favors 3D growth of thyroid cancer cells and that IL-6 plays an important role in this process. Il-6 favoring MCS formation and cell aggregation at 1*g* (liquid overlay technique) and simulated microgravity was elevated in simulated microgravity produced on the RPM or CLINO, which is in contrast to experiments in space using the FTC-133 cell line [22]. In addition, also IL-8 exerted beneficial effects on spheroid formation under 1g-conditions. This finding has to be investigated in more detail in future s-μg experiments. Cell line-specific differences between ML-1 and RO82-W-1 were found for the release of MCP-1. Therefore, further studies are required to elaborate possible relationships between the differentiation of cancer cells and gravity-dependent changes of cells.

## Conclusions

Taken together, the results obtained in this study reveal that experiments with the RPM and the CLINO show similar effects on ML-1 and RO82-W-1 cells with regard to cytokine release and expression of cytoskeletal proteins. Only in 2 out of 14 cytokines released by ML-1 cells and measured by Multianalyte Profiling technology, we found significantly different effects exerted by the CLINO in comparison to the RPM. Hence, the presented results demonstrate a number of common effects of the two devices on the cells. This makes simulation of microgravity on Earth to a valuable method, if one takes in mind that the simulations have to be verified under real microgravity conditions. Experiments in microgravity provide an interesting tool for exploring new targets for cancer therapy. Staying critical and taking side effects into account, ground-based facilities could provide cheaper alternatives of spaceflight research at least for studies on dedicated aspects. The signaling pathways resulting in microgravity-related alterations have to be determined [59] in order to identify and separate distinct proteins playing a role in adaption to microgravity and to cancer progression.

## References

[1] White RJ, Averner M. Humans in Space. Nature. 2001;409: 1115–1118. 11234026 10.1038/35059243

[2] Hughes-Fulford M. To infinity…and beyond! Human spaceflight and life science. FASEB J. 2011;25: 2858–2864. 10.1096/fj.11-0902ufm 21880668 PMC6188462

[3] Pietsch J, Bauer J, Egli M, Infanger M, Wise P, Ulbrich C, et al The effects of weightlessness on the human organism and mammalian cells. Curr Mol Med. 2011;11: 350–364. 21568935 10.2174/156652411795976600

[4] Grimm D, Wise P, Lebert M, Richter P, Baatout S. How and why does the proteome respond to microgravity? Expert Rev Proteomics. 2011;8: 13–27. 10.1586/epr.10.105 21329425

[5] Hammond TG, Benes E, O’Reilly KC, Wolf DA, Linnehan RM, Taher A, et al Mechanical culture conditions effect gene expression: gravity-induced changes on the space shuttle. Physiol Genomics. 2000;3: 16–173. 10.1152/physiolgenomics.2000.3.3.163 11015612

[6] Griffoni C, Di Molfetta S, Fantozzi L, Zanetti C, Pippia P, Tomasi V, et al Modification of proteins secreted by endothelial cells during modeled low gravity exposure. J Cell Biochem. 2011;112: 265–272. 10.1002/jcb.22921 21069737

[7] Grimm D, Pietsch J, Wehland M, Richter P, Strauch SM, Lebert M, et al The impact of microgravity-based proteomics research. Expert Rev. Proteomics. 2014;11: 465–476. 10.1586/14789450.2014.926221 24957700

[8] Maccarrone M, Battista N, Meloni M, Bari M, Galleri G, Pippia P, et al Creating conditions similar to those that occur during exposure of cells to microgravity induces apoptosis in human lymphocytes by 5-lipoxygenase-mediated mitochondrial uncoupling and cyto- chrome c release. J Leukoc Biol. 2003;73, 472–481. 12660222 10.1189/jlb.0602295

[9] Uva BM, Masini MA, Sturla M, Bruzzone F, Giuliani M, Tagliafierro G, et al Microgravity-induced apoptosis in cultured glial cells. Eur J Histochem. 2002;46, 209–214. 12472115 10.4081/1681

[10] Boonyaratanakornkit JB, Cogoli A, Li CF, Schopper T, Pippia P, Galleri G, et al Key gravity-sensitive signaling pathways drive T cell activation. FASEB J. 2005;19, 2020–2022. 16210397 10.1096/fj.05-3778fje

[11] Buravkova LB, Romanov YA. The role of cytoskeleton in cell changes under condition of simulated microgravity. Acta Astronaut. 2001;48: 647–650. 11858272 10.1016/s0094-5765(01)00023-6

[12] Kumei Y, Morita S, Katano H, Akiyama H, Hirano M, Oyha K, et al Microgravity signal ensnarls cell adhesion, cytoskeleton, and matrix proteins of rat osteoblasts Ann. N.Y. Acad. Sci. 2006;1090: 311–317. 10.1196/annals.1378.034 17384275

[13] Grimm D, Bauer J, Kossmehl P, Shakibaei M, Schönberger J, Pickenhahn H, et al Simulated microgravity alters differentiation and increases apoptosis in human follicular thyroid carcinoma cells. FASEB J. 2002;16: 604–606. 11919168 10.1096/fj.01-0673fje

[14] Grimm D, Wehland M, Pietsch J, Aleshcheva G, Wise P, Van Loon J, et al Growing tissues in real and simulated microgravity: New methods for tissue engineering. Tissue Engineering—Part B: Reviews. 2014;20: 555–566. 24597549 10.1089/ten.teb.2013.0704 PMC4241976

[15] Becker JL, Souza GR. Using space-based investigations to inform cancer research on Earth. Nat Rev Cancer. 2013;13: 315–327. 10.1038/nrc3507 23584334

[16] Pietsch J, Ma X, Wehland M, Aleshcheva G, Schwarzwälder A, Segerer J, et al Spheroid formation of human thyroid cancer cells in an automated culturing system during the Shenzhou-8 space mission. Biomaterials. 2013;34: 7694–7705. 10.1016/j.biomaterials.2013.06.054 23866977

[17] Pietsch J, Kussian R, Sickmann A, Bauer J, Weber G, Nissum M, et al Application of free-flow IEF to identify protein candidates changing under microgravity conditions. Proteomics. 2010;10: 904–913. 10.1002/pmic.200900226 20049858

[18] Warnke E, Pietsch J, Wehland M, Bauer J, Infanger M, Görög M, et al Spheroid formation of human thyroid cancer cells under simulated microgravity: a possible role of CTGF and CAV1. Cell Commun Signal. 2014;12: 32 10.1186/1478-811X-12-32 24885050 PMC4020378

[19] Grosse J, Wehland M, Pietsch J, Schulz H, Saar K, Hübner N, et al Gravity-sensitive signaling drives 3-dimensional formation of multicellular thyroid cancer spheroids. FASEB J. 2012;26: 5124–5140. 10.1096/fj.12-215749 22964303

[20] Ma X, Wehland M, Aleshcheva G, Hauslage J, Waßer K, Hemmersbach R, et al Interleukin-6 expression under gravitational stress due to vibration and hypergravity in follicular thyroid cancer cells. PLoS ONE. 2013;8: e68140 10.1371/journal.pone.0068140 23844163 PMC3699536

[21] Pietsch J, Sickmann A, Weber G, Bauer J, Egli M, Wildgruber R, et al A proteomic approach to analysing spheroid formation of two human thyroid cell lines cultured on a random positioning machine. Proteomics. 2011;11: 2095–2104. 10.1002/pmic.201000817 21520503

[22] Ma X, Pietsch J, Wehland M, Schulz H, Saar K, Hübner N, et al Differential gene expression profile and altered cytokine secretion of thyroid cancer cells in space. FASEB J. 2014;28: 813–835. 10.1096/fj.13-243287 24196587

[23] Riwaldt S, Pietsch J, Sickmann A, Bauer J, Braun M, Segerer J, et al Identification of proteins involved in inhibition of spheroid formation under microgravity. Proteomics. 2015 4 29. [Epub ahead of print] 10.1002/pmic.201500067 25930030

[24] Grimm D, Bauer J, Kromer E, Steinbach P, Riegger G, Hofstädter F. Human follicular and papillary thyroid carcinoma cells interact differently with human venous endothelial cells. Thyroid. 1995;5: 155–164. 7580262 10.1089/thy.1995.5.155

[25] Schönberger J, Bauer J, Spruß T, Weber G, Chahoud I, Eilles C, et al Establishment and characterization of the follicular thyroid carcinoma cell line ML-1. J Mol Med. 2000;78: 102–110. 10794546 10.1007/s001090000085

[26] Estour B, Van Herle AJ, Juillard GJ, Totanes TL, Sparkes RS, Giuliano AE, Klandorf H. Characterization of a human follicular thyroid carcinoma cell line (UCLA RO 82 W-1). Virchows Arch B Cell Pathol Incl Mol Pathol. 1989;57: 167–174. 2570483 10.1007/BF02899078

[27] Rochfort KD, Cummins PM. Cytokine-mediated dysregulation of zonula occludens-1 properties in human brain microvascular endothelium. Microvasc Res. 2015;100: 48–53. 10.1016/j.mvr.2015.04.010 25953589

[28] Sun Q, Sun F, Wang B, Liu S, Niu W, Liu E, Peng C, Wang J, Gao H, Liang B, Niu Z, Zou X, Niu J. Interleukin-8 promotes cell migration through integrin αvβ6 upregulation in colorectal cancer. Cancer Lett. 2014;354: 245–53. 10.1016/j.canlet.2014.08.021 25150782

[29] Li X, Xu M, Liu M, Ji Y, Li Z. TNF-alpha and IL-6 inhibit apolipoprotein A-IV production induced by linoleic acid in human intestinal Caco2 cells. J Inflamm (Lond). 2015;12: 22. 25861245 10.1186/s12950-015-0069-0 PMC4389805

[30] van Loon JJWA. Some history and use of the random positioning machine, RPM, in gravity related research. Advances in Space Research. 2007;39: 1161–1165.

[31] Eiermann P, Kopp S, Hauslage J, Hemmersbach R, Gerzer R, Ivanova K: Adaptation of a 2D clinostat for simulated microgravity experiments with adherent cells. Micrograv Sci Technol. 2013;25: 153–159.

[32] Ulbrich C, Westphal K, Pietsch J, Winkler HD, Leder A, Bauer J, et al Characterization of human chondrocytes exposed to simulated microgravity. Cell Physiol Biochem. 2010;25: 551–560. 10.1159/000303059 20332636

[33] Riecke K, Grimm D, Shakibaei M, Kossmehl P, Schulze-Tanzil G, Paul M, et al (2002) Low doses of 2,3,7,8-tetrachlorodibenzo- p-dioxin increase transforming growth factor beta and cause myocardial fibrosis in marmosets (Callithrix jacchus). Arch Tox. 2002;76: 360–366. 10.1007/s00204-002-0338-6 12107654

[34] Rothermund L, Kreutz R, Kossmehl P, Fredersdorf S, Shakibaei M, Paul M, et al Early onset of chondroitin sulfate and osteopontin expression in angiotensin II dependent left ventricular hypertrophy. Am J of Hypertens. 2002;15: 644–652. 12118914 10.1016/s0895-7061(02)02956-4

[35] Ma X, Wildgruber R, Bauer J, Weber G, Infanger M, Grosse J, et al The use of sigmoid pH gradients in free-flow isoelectric focusing of human endothelial cell proteins. Electrophoresis. 2012;33: 1349–1355. 10.1002/elps.201100598 22648801

[36] Aleshcheva G, Sahana J, Ma X, Hauslage J, Hemmersbach R, Egli M, et al Changes in morphology, gene expression and protein content in chondrocytes cultured on a random positioning machine. PLoS One. 2013;8: e79057 10.1371/journal.pone.0079057 24244418 PMC3823937

[37] Aleshcheva G, Wehland M, Sahana J, Bauer J, Corydon TJ, Hemmersbach R, et al Moderate alterations of the cytoskeleton in human chondrocytes after short-term microgravity produced by parabolic flight maneuvers could be prevented by up-regulation of BMP-2 and SOX-9. FASEB J. 2015; 29: 2303–2014. 10.1096/fj.14-268151 25681461

[38] Wehland M, Aleshcheva G, Schulz H, Saar H, Hübner N, Hemmersbach R, et al Differential gene expression of human chondrocytes cultured under short-term altered gravity conditions during parabolic flight maneuvers. Cell Commun Signal. 2015;13:18 10.1186/s12964-015-0095-9 25889719 PMC4369370

[39] Versari S, Villa A, Bradamante S, Maier JA. Alterations of the actin cytoskeleton and increased nitric oxide synthesis are common features in human primary endothelial cell response to changes in gravity. Biochim Biophys Acta. 2007;1773: 1645–1652. 17609119 10.1016/j.bbamcr.2007.05.014

[40] Infanger M, Ulbrich C, Baatout S, Wehland M, Kreutz R, Bauer J, et al Modeled gravitational unloading induced downregulation of endothelin-1 in human endothelial cells. J Cell Biochem. 2007;101: 1439–1455. 17340622 10.1002/jcb.21261

[41] Grimm D, Bauer J, Ulbrich C, Westphal K, Wehland M, Infanger M, et al Different responsiveness of endothelial cells to vascular endothelial growth factor and basic fibroblast growth factor added to culture media under gravity and simulated microgravity. Tissue Eng Part A. 2010;16: 1559–1573. 10.1089/ten.TEA.2009.0524 20001221

[42] Herranz R, Anken R, Boonstra J, Braun M, Christianen PC, de Geest M, et al Ground-based facilities for simulation of microgravity: organism-specific recommend-dations for their use, and recommended terminology. Astrobiology. 2013;13: 1–17. 10.1089/ast.2012.0876 23252378 PMC3549630

[43] Zeng J, Xie K, Wu H, Zhang B, Huang C. Identification and functional study of cytokines and chemokines involved in tumorigenesis. Comb. Chem. High Throughput Screen. 2012;15: 276–285. 22221060 10.2174/138620712799218608

[44] Todaro M, Zerilli M, Ricci-Vitiani L, Bini M, Perez Alea M, Maria Florena A, et al Autocrine production of interleukin-4 and interleukin-10 Is required for survival and growth of thyroid cancer cells. Cancer Res. 2006;66: 1491–1499. 16452205 10.1158/0008-5472.CAN-05-2514

[45] Provatopoulou X, Georgiadou D, Sergentanis TN, Kalogera E, Spyridakis J, Gounaris A, et al Interleukins as markers of inflammation in malignant and benign thyroid disease—Inflamm Res. 2014;63: 667–674. 10.1007/s00011-014-0739-z 24794392

[46] Deleuran B, Lemche P, Kristensen M, Chu CQ, Field M, Jensen J, et al Localisation of interleukin 8 in the synovial membrane, cartilage- pannus junction and chondrocytes in rheumatoid arthritis. Scand J Rheumatol. 1994;23: 2–7. 8108662 10.3109/03009749409102126

[47] Terada H, Urano T, Konno H. Association of interleukin-8 and plasminogen activator system in the progression of colorectal cancer. Eur Surg Res. 2005;37: 166–172. 16088182 10.1159/000085964

[48] Lee YC, Chung JH, Kim SK, Rhee SY, Chon S, Oh SJ, et al Association between interleukin 17/interleukin 17 receptor gene polymorphisms and papillary thyroid cancer in Korean population. Cytokine. 2015;71: 283–288. 10.1016/j.cyto.2014.11.011 25484349

[49] Tuttle RM, Fleisher M, Francis GL, Robbins RJ. Serum vascular endothelial growth factor levels are elevated in metastatic differentiated thyroid cancer but not increased by short-term TSH stimulation. J Clin Endocrinol Metab. 2002;87: 1737–1742. 11932308 10.1210/jcem.87.4.8388

[50] Grimm D, Bauer J, Schoenberger J. Blockade of neoangiogenesis, a new and promising technique to control the growth of malignant tumors and their metastases. Curr Vasc Pharmacol. 2009;7: 347–357. 19601859 10.2174/157016109788340640

[51] Kristensen TB, Knutsson ML, Wehland M, Laursen BE, Grimm D, Warnke E, et al Anti-vascular endothelial growth factor therapy in breast cancer. Int J Mol Sci. 2014;15: 23024–23041. 10.3390/ijms151223024 25514409 PMC4284752

[52] Wehland M, Bauer J, Infanger M, Grimm D. Target-based anti-angiogenic therapy in breast cancer. Curr Pharm Des. 2012;18: 4244–4257. 22632607 10.2174/138161212802430468

[53] Balkwill F, Gopinathan G, Milagre C, Pearce OM, Reynolds LE, Hodivala-Dilke K, et al Interleukin-6 stimulates defective angiogenesis. Cancer Res. 2015 6 16. [Epub ahead of print] 10.1158/0008-5472.CAN-15-1227 PMC4527186 26081809

[54] Tanaka K, Kurebayashi J, Sohda M, Nomura T, Prabhakar U, Yan L, et al The expression of monocyte chemotactic protein-1 in papillary thyroid carcinoma is correlated with lymph node metastasis and tumor recurrence. Thyroid. 2009;19: 21–25. 10.1089/thy.2008.0237 19072670

[55] Ulbrich C, Pietsch J, Grosse J, Wehland M, Schulz H, Saar K, et al Differential gene regulation under altered gravity conditions in follicular thyroid cancer cells: relationship between the extracellular matrix and the cytoskeleton. Cell Physiol Biochem. 2011;28: 185–198. 10.1159/000331730 21865726

[56] Grosse J, Wehland M, Pietsch J, Ma X, Ulbrich C, Schulz H, et al Short-term weightlessness produced by parabolic flight maneuvers altered gene expression patterns in human endothelial cells. FASEB J. 2012;26: 639–655. 10.1096/fj.11-194886 22024737

[57] Lewis ML, Reynolds JL, Cubano LA, Hatton JP, Lawless BD, Piepmeier EH. Spaceflight alters microtubules and increases apoptosis in human lymphocytes (Jurkat). FASEB J. 1998;12: 1007–1018. 9707173 10.1096/fasebj.12.11.1007

[58] Xu J, Zhang Z, Chen J, Liu F, Bai L. Overexpression of β-actin is closely associated with metastasis of gastric cancer. Hepatogastroenterology. 2013;60: 620–623. 23635433 10.5754/hge11038

[59] Pietsch J, Riwaldt S, Bauer J, Sickmann A, Weber G, Grosse J, et al Interaction of proteins identified in human thyroid cells. Int J Mol Sci. 2013;14: 1164–1178. 10.3390/ijms14011164 23303277 PMC3565314

[60] Ensinger C, Obrist P, Bacher-Stier C, Mikuz G, Moncayo R, Riccabona G. β1-Integrin expression in papillary thyroid carcinoma. Anticancer Res 1998;18: 33–40. 9568052

[61] Demeure MJ, Damsky CH, Elfman F, Goretzki PE, Wong MG, Clark OH. Invasion by cultured human follicular thyroid cancer correlates with increased β1 integrins and production of proteases. World J Surg. 1992;16: 770–776. 1384245 10.1007/BF02067383

[62] Vignoud L, Albigès-Rizo C, Frachet P, Block MR. NPXY motifs control the recruitment of the α5β1 integrin in focal adhesions independently of the association of talin with the β1 chain. J Cell Sci. 1997;110: 1421–1430. 9217328 10.1242/jcs.110.12.1421

[63] Sakamoto S, McCann RO, Dhir R, Kyprianou N. Talin1 promotes tumor invasion and metastasis via focal adhesion signaling and anoikis resistance. Cancer Res. 2010;70: 1885–1895. 10.1158/0008-5472.CAN-09-2833 20160039 PMC2836205

[64] Infanger M, Kossmehl P, Shakibaei M, Schulze-Tanzil G, Cogoli A, Faramarzi S, et al Longterm conditions of mimicked weightlessness influences the cytoskeleton in thyroid cells. J Gravit Physiol. 2004;11: P169–P172. 16237826

